# Bio-Stimulated Surface Healing of Historical and Compatible Conservation Mortars

**DOI:** 10.3390/ma16020642

**Published:** 2023-01-09

**Authors:** Snežana Vučetić, Damir Čjepa, Bojan Miljević, John Milan van der Bergh, Olja Šovljanski, Ana Tomić, Emilija Nikolić, Siniša Markov, Helena Hiršenberger, Jonjaua Ranogajec

**Affiliations:** 1 University of Novi Sad, Laboratory for Materials in Cultural Heritage, Faculty of Technology, Bulevar Cara Lazara 1, 21000 Novi Sad, Serbia; 2 La Farge Serbia, Holcim Group, Trg Beocinske Fabrike Cementa 1, 21300 Beočin, Serbia; 3 Built Environment and Sustainable Technologies (BEST) Research Institute, Liverpool John Moores University, 15-21 Webster St., Liverpool L3 2ET, UK; 4 Institute of Archaeology, Kneza Mihaila 35/IV, 11000 Belgrade, Serbia; 5 FEFA Faculty, Metropolitan University, Bulevar Zorana Djindjića 44, 11070 Belgrade, Serbia

**Keywords:** bio-stimulated healing, lime mortar conservation, compatible mortars, precipitation into cracks, microbiologically induced calcium carbonate precipitation (MICP)

## Abstract

The main focus of this research was the bio-stimulated healing of cracks in lime mortar samples (historical and newly designed). The investigation started from comprehensive characterisation of historical mortars, while in the next stage a compatible conservation mortar was designed and characterised, with special attention given to the contact zone formation between original and conservation mortars. The next step was the design of a bio-stimulating crack-sealing agent, a two-component liquid system: bacteria culture *Sporosarcina pasteurii* DSM 33 and nutrients. Both historical and conservation mortar samples were used in order to study their potentials for bio-stimulated surface-crack repair. The experiment lasted for 150 days, allowing the ureolytic bacteria *Sporosarcina pasteurii* DSM 33 to induce the precipitation of calcium carbonate into cracks and heal the damaged surface of the tested materials. The healing phenomenon was continuously monitored during a period of 150 days. Special attention was given to the evaluation of the morphology, chemical and structural characteristics of the deposits created in/on the surface cracks, monitored by optical microscopy, SEM, XRF and XRD analyses. The obtained results present valuable input for the application of the developed system in real environmental conditions as a solution for the future sustainable architectural conservation of traditionally prepared mortars.

## 1. Introduction

Ageing and degradation of mortar are considered to be some of the most significant challenges for conservation scientists and practitioners in the field of architectural heritage. Having a thorough understanding of the materials and technologies used in historical buildings is crucial for creating sustainable preservation strategies. Conservation mortars in particular must meet strict chemical, mineralogical, textural and mechanical compatibility requirements. The approach to the healing of historical and conservation mortars might be sought in the use of advanced functional materials. They can provide the repair of functional properties and aesthetic appearances coupled with long-lasting surface protection from weathering and microbiological corrosion [1].

In the period from 2020 to 2022, a scientific and research project, Mortar Design for Conservation—Danube Roman Frontier 2000 Years After (MoDeCo2000), was conducted. During the project, more than 120 samples of mortars dated to the period from the first to the sixth century were analysed, with an aim to develop compatible mortars for the buildings from which they originated. After the field research and sampling of mortars from 40 buildings (24 archaeological sites and monuments of culture), many conclusions on the common use of raw materials and production technologies were made. These objects were placed along the part of the Roman Danube Limes in modern-day Serbia, which is currently on the UNESCO tentative list Frontiers of the Roman Empire—The Danube Limes (Serbia) [2,3]. The results were further used for the development of models of conservation mortars. The research showed that a common methodology for the research of historical and the preparation of conservation mortars for the buildings of The Danube Limes in Serbia could be proposed as an important set of recommendations for the protection of these monuments. The application of a conservation mixture for a bedding mortar, made according to the characteristics of the promising conservation models developed in the laboratory, was conducted on the wall of a later Roman tomb of Brestovik [4]. The applied lime mortar was made of river aggregate from the Danube, slaked lime and clay. Although no large cracks were developed after the application, it was clear that the applied surface was very small and that only after the application on a larger area could the behaviour of the mortar in real conditions be adequately conducted. Additionally, it is very common for cracks to appear in historical mortars due to different external or internal influences (including conservation procedures using incompatible materials), but also it could be the consequences of curing process. The MoDeCo2000 project offered recommendations for the research of historical mortars and the design of compatible conservation mortars, but the experimental application of conservation mixtures was conducted only on chosen monuments with the aim of gaining appropriate conclusions that can be used for the overall fund of Roman monuments along the Danube in Serbia. However, dozens of developed conservation mixtures, applied and artificially aged in the laboratory for many historic mortars, were recommended to be further applied and monitored. Thus, we wanted to make one step in advance and offer solutions for the problems that could appear with mortars in future experiments on-site. Additionally, our goal was to solve the problems that historical mortars on the Danube Limes already have. In collaboration with other researchers, methods of crack repair were investigated since surface cracks are the most common problem that conservators encounter during the application of new mortars. In this paper, research on the bio-stimulated surface healing of newly developed conservation mortars was presented, but for the understanding of the overall process, historical mortars were investigated as well. Since the experiment of bio-healing takes a significantly long period compared to the duration of the MoDeCo2000 project, it was decided to research its effects on an already-developed case study of conservation mortar samples that has been tested on large historical surfaces (in situ), that is, another building on the Danube, the Bač Fortress, which also aspires to be a part of the UNESCO World Heritage List [3].

In recent years, a number of studies have explored the use of bacteria cultures with bio-calcification potential to induce a self-healing effect in cement materials [1]. However, only a few publications have considered this approach for the healing of historical lime-based mortars or compatible conservation mortars [5]. The interest of researchers and conservators for this topic is increasing due to an emerging awareness of its beneficial potential in both modern and historical architecture. Historic renders and mortars often show diffused crack patterns caused by different construction techniques and loading histories [1,5]. Under certain conditions, mortars and concrete can have the ability to heal naturally or “autogenously.” Some methods also introduce “autonomic” healing. The effectiveness of this healing process is influenced by several factors, including humidity and temperature, the pH value of the structure matrix, the presence of dissolved inorganic carbon and calcium ions and the age and level of damage to the structure. Following these parameters, engineered healing based on microbiologically induced calcium carbonate precipitation (MICP) presents a promising approach, which is not widely used in case of conservation mortars. The use of bacteria to induce the precipitation of calcium carbonate has great potential for fast and effective crack repair. This process is not only environmentally friendly but also provides compatibility and strong bonding between the matrix and the repair material [6,7]. When bacteria with bio-calcifying potential are incorporated into the matrix or onto the surface, the viable bacterial cells can activate the healing process by the precipitation of inorganic minerals.

The approach of bacteria-induced healing could be used for surface cracks in historical monuments and buildings as well as for the development of self-healing biomortar and bioconcrete [8]. The used bacteria induce the precipitation of carbonaceous minerals through metabolic processes such as organic acid utilisation, urea hydrolysis and denitrification [9]. The metabolic processes of certain bacteria can alter the pH value or the concentration of dissolved inorganic deposits in the matrix. It is thought that the walls of these bacteria serve as nucleation sites for crystals in a supersaturated matrix of calcium and carbonate ions. Additionally, these bacteria can change the saturation state of an undersaturated solution by catalysing the formation of minerals [10]. Due to their ability to tolerate alkaline conditions and produce high levels of the enzyme urease, certain bacteria, such as *Sporosarcina pasteurii*, *Sporosarcina urea*, *Bacillus sphaericus* and *Bacillus megaterium*, have been extensively tested for their use in improving the self-healing capacity of porous and cracked concrete. These bacteria have been applied to cementitious materials for this purpose. It is believed that ureolytic bacteria can use the enzyme urease to induce the formation of one mole of calcium carbonate (CaCO_3_) through the metabolism of one mole of urea. [11]. As an example, under optimal conditions (108 cfu/mL, 28 °C, 1M urea, 20 g/L yeast extract, 1M Ca^2+^), *B. sphaericus* cells were able to produce 60 g of calcium carbonate (CaCO_3_) within a single day [12]. In comparison, *S. pasteurii* needed less than 24 h to convert calcium and urea into CaCO_3_ [13]. The method of applying the bacterial suspension and nutrients into cracks is also very important. So far, the external healing of surface cracks has largely been limited to porous limestone, where the surface deposition of calcium carbonate has reduced water permeability by 65% to 90%, depending on the newly formed porosity [14].

The efficiency of the bacterial treatment of surface cracks largely depends on crack width, curing conditions and the age of the substrate. Within the study presented in this paper, based on the in-depth characterization of original samples of historical mortar, a conservation mortar was designed. Compatible conservation mortars are generally case-specific; they have to comply with the performance of the historical mortar and with compatibility requirements. The characteristics of raw materials are the most influential parameters for the modification and optimization of their performance [15]. Angular coarse aggregates, which offer a larger surface area, are more effective than smooth coarse aggregates for healing purposes [5], something that was taken into account in this paper regarding the design of conservation mortar with healing potential. Furthermore, as far as we know, the evolution of contact zone formation between original and new conservation mortars has not been presented in the literature yet.

Additionally, the healing potential also depends on the mortar age at the time of the already existing cracks. On-going hydration and carbonation are the dominant factors in the case of “young” mortars due to unhydrated components, while CaCO_3_ precipitation becomes more important with increasing age in humid environments [16]. In this study, a bacteria-based system for external bio-stimulated crack closure was developed in the laboratory. The system was a two-component healing agent (bacteria and nutrients). To test its potential to repair mortar surfaces, this system was applied onto/into the existing cracks of historical mortar samples as well as onto/into artificially produced surface cracks of conservation mortar models. The healing phenomenon was continuously monitored during a period of 150 days. Special attention was given to the evaluation of the morphology, chemical and structural characteristics of the deposits created in/on the surface cracks, monitored by optical microscopy, SEM, XRF and XRD analyses.

## 2. Materials and Methods

**Figure 1:**
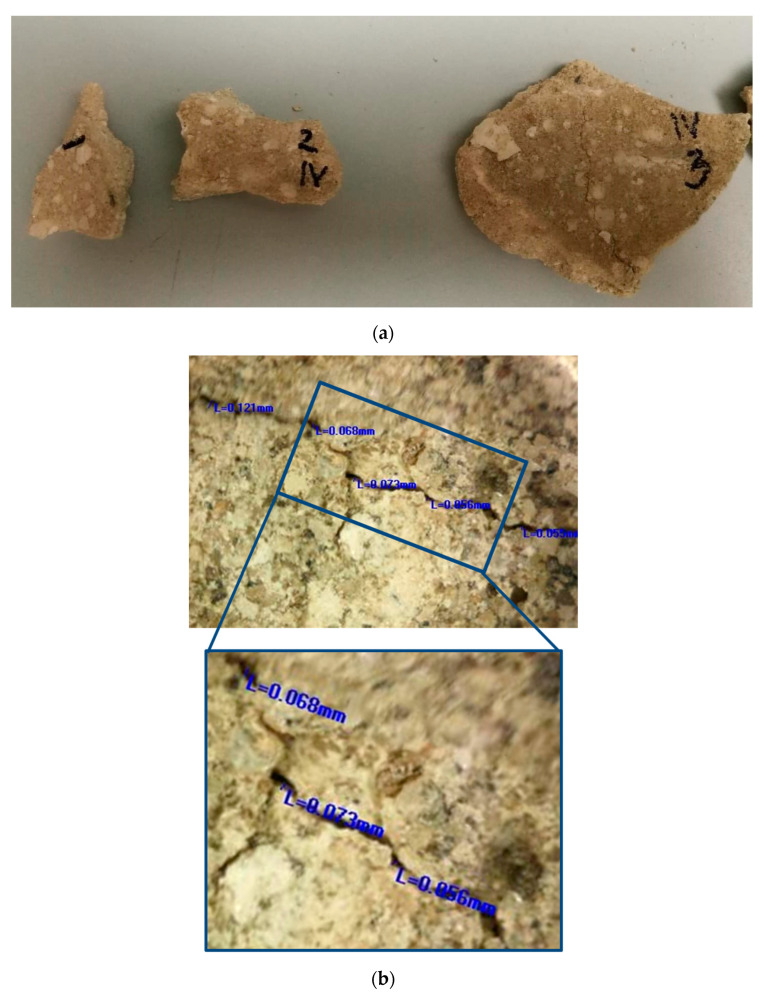
Original mortar: (**a**) photo of the sampled materials; (**b**) example of microscopic images of the cracks in original walls (measured crack width: 0.121 mm; 0.068 mm; 0.073 mm; 0.056 mm; 0.059 mm; average 0.075 mm).

**Figure 2:**
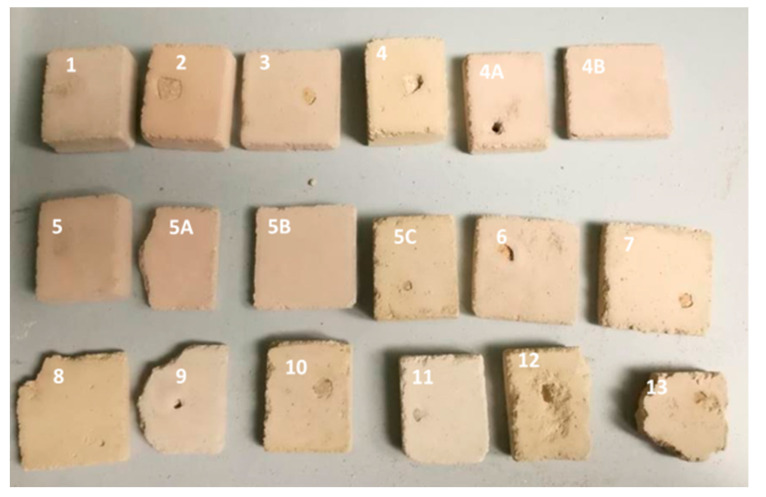
Photo of the laboratory-prepared MM samples after artificial ageing, Table 1.

This research involved the use of two types of mortars: (1) samples of historical mortars (HM) and (2) laboratory-prepared conservation mortars (MM) intended for future use on historical sites, as shown in Figure 1 and Figure 2. Both groups of mortars were treated with a bio-activated healing agent, and the effectiveness of this treatment was examined.

### 2.1. Characterisation of Historical Mortar Samples

Historical mortar samples were taken from the medieval Bač Fortress located in northern Serbia (cultural landscape of Bač and its surroundings, submitted by: Permanent Delegation of Serbia to UNESCO, Autonomous Province of Vojvodina; coordinates: E19 52 57 N45 23 31; Ref.: 6386) [3]. The Bač Fortress is an authentic “water town/burg”, designed as a defence system and adapted to marshy land, which is quite unique among the fortifications on the left bank of the Danube River. The fortified castle was built between 14th and 16th centuries, and it was listed as a protected monument in 1948. Today, Bač Fortress is a symbol of the local identity and is recognised as a carrier of multiple values. Together with the educational centre that was built in the suburbs, it has also become a centre where professional knowledge about heritage conservation and management is gained, enhanced and shared, and is a place of culture and creativity, where local communities and visitors can interact. We chose this object based on several reasons. First of all, our team already had significant experience in the development of and recommendations of instructions for the conservation of the finishing floor mortars of the inner part of the dungeon tower of Bač Fortress [6]. In addition, at the time of study, conservation works were ongoing, and a large number of render mortar samples were available for our experiment. The most important fact was that we planned these experiments for several years in order to investigate the application of this research in real environmental conditions. Namely, based on our agreement with conservators, our plan was to use results of this research on the inner walls of Bač Fortress (after the conservation).

More than 30 small samples (approximately several cm in diameter) with irregular shapes and a diversity of surface cracks with varieties in their width were sampled, as shown in Figure 1a. These samples were the materials that the conservators decided to remove prior to the conservation works because they were in a very bad condition (some parts of the walls were still covered with this mortar). In order to have as realistic as possible of an approach for the bio-stimulated healing experiment, the samples were grouped based on their crack widths. The samples which were too small or unusable for the crack-healing investigation were used for other laboratory investigations. Laboratory characterisation of the historical mortar samples was performed using sieving and chemical analysis, stereo-optic microscopy, X-ray fluorescence (XRF), X-ray diffraction (XRD) and scanning electron microscopy (SEM) coupled with energy-dispersive spectroscopy (EDS). Sieving and chemical analysis (acid dissolution) of the historical samples were conducted in accordance with RILEM recommendations in order to determine the aggregate and binder ratio [6]. The basic morphological features of the historical samples were determined by optical microscopy (OMANO OMXTL/V7 Articulated Boom Microscope, Microscope LLC, Roanoke, VA, USA). The mineralogical composition of the powdered samples after sieving analysis (binder (fraction < 63 µm), agglomerated binder/lumps (fraction > 710 µm) and aggregate (710–63 µm)) was determined by XRD analysis with a PHILIPS PW 1050 Diffractometer (Philips, Netherlands) with a Cu-K_α_ radiation source. The elemental analysis (XRF) was performed directly on the samples’ surfaces without any preparation. These measurements were performed with a BRUKER μXRF ARTAX 200 system (Bruker Nano GmbH, Berlin, Germany) using a Rh radiation source, 25 kV and 1.5 mA under a He atmosphere. SEM measurements were performed after 28 days of incubation using a JSM-5500LV (JEOL, Tokyo, Japan) device and after 150 days of incubation using a JSM-6460LV (JEOL, Tokyo, Japan) device. None of the examined samples were previously sputter-coated with gold.

### 2.2. Design of Conservation Mortars

The composition and production technology for the conservation model mortars (intended for future use as a rendering layer) were developed based on the characteristics of the historical mortars (HM samples). For the design of compatible systems, an already-proven methodology for the design of compatible mortars for a late Roman tomb in Serbia was used [4]. The designed model mortars were artificially aged in the laboratory using a Binder Climate Chamber KBWF 240 device. Their compatibility was compared to that of the historical mortars in terms of their colour and mechanical properties. The mechanical properties were measured using a Drilling Resistance Measuring System (DRMS, SINT Technology, Italy), and the colour characteristics were measured with a CM-700D spectrophotometer (Konica Minolta, Tokyo, Japan) and calculated in the CIE colour space [17,18]. The interaction between the prepared model mortars and the original historical samples was studied using a stereo-optical microscope.

The selected components for the conservation mortar mixture were as follows: lime, crushed brick as pozzolanic material, river sand and milled limestone as aggregates and water. A total of 18 different mixtures of conservation mortar (MM) were prepared in the laboratory, with variations in type and the percentage of aggregate components, as shown in Table 1. To allow an evaluation of the compatibility between the original historical mortars and the laboratory-designed conservation mortars, small fragments of the HM sample mortar were put inside one set of the fresh MM samples. After a defined time, the formed contacts were analysed (Figure 2). The composition of this mortar mixture was as follows: binder-to-aggregate volume ratio of 1 to 2 (slaked lime 1, crushed brick 0.2 as pozzolanic material, river sand 1.6 and milled limestone 0.2).

**Figure 3:**
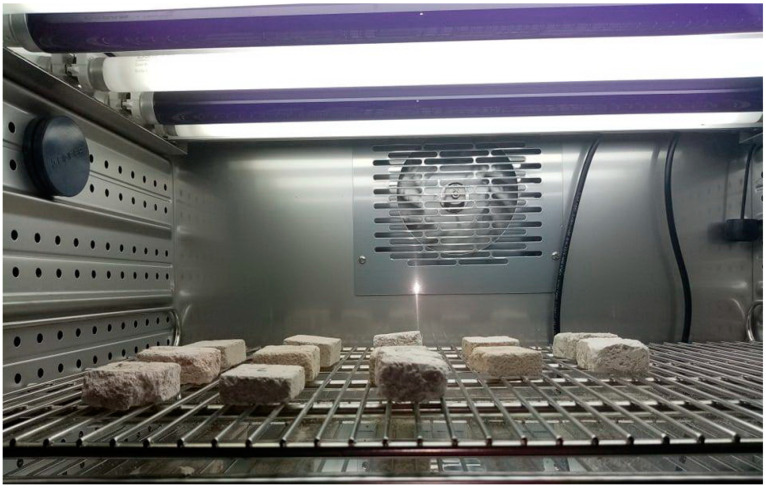
Artificial ageing of the MM mortar samples in the chamber.

The artificial ageing procedure was set up based on the average climatic parameters in Serbia, relevant scientific publications, our previous experience and an already proven methodology for the design of compatible systems [4,6]. In the first 5 days, the MM samples were exposed to room conditions (T = 20 °C and RH = 60%), after which they were placed in a climate chamber for one month, combining exposure to high and low temperatures and different relative humidities (T = 30 °C and RH = 80% for 5 days, T = 0 °C and RH = 60% for 24 h, repeated for 5 cycles in total). Then, the MM samples were again subjected to room conditions for 7 days. After the artificial ageing in laboratory (Figure 3), the MM samples were analysed.

After the artificial ageing of the conservation mortar models, a system of surface cracks was designed. A drilling resistance measuring system was used for this purpose (average force was 0.3 N). The samples were drilled on their side until a crack on the surface occurred. The cracks were of an irregular shape, non-uniform in width and depth, aiming to imitate a realistic mechanical deformation in mortars. Only the samples with surface cracks which corresponded to the type and dimension of the cracks studied on the historical mortar surfaces were used, as shown in Figure 1. Namely, after the evaluation of the crack width on the walls (digital optical microscopy ViTiny, VT-300; Imaging Analysis Software), historical samples and samples of model mortars were chosen for further experiments. The samples with an initial crack width nearly equal to or higher than that of the cracks on the original surfaces were chosen (samples with an average crack width of 0.07–0.09 mm).

### 2.3. Design of Bio-Stimulated Healing Process

Due to its significant role in the MICP process, *Sporosarcina pasteurii* has been widely used in creating model systems of bacteria-induced calcite precipitation in extreme environmental conditions. This sporogenic bacterium is well known in various engineered-healing processes and has an optimal pH value for growth and urease activity between 7.5 and 9 [19,20]. In this experiment, *S. pasteurii* DSM 33 (German Collection of Microorganisms and Cell Cultures) was chosen to induce the precipitation of calcium carbonate for the repair of surface cracks. The inoculum was prepared by aerobically incubating *S. pasteurii* DSM 33 on Trypton Soya Agar (TSA, HiMedia, India) with the addition of 20% urea (Difco, HiMedia, USA) at 30 °C for 6 days. The initial number of viable spores was estimated after thermal treatment (80 °C, 10 min) by counting colonies after incubation at 30 °C for 48 h on TSA with addition of 20% urea. A modified urea-based broth (consisting of nutrient broth 30 g/L, NH_4_Cl_2_ 100 g/L, NaHCO_3_ 21.2 g/L and urea 200 g/L) was used as the nutrient medium for the MICP process.

In order to ensure the activity of the ureolytic bacteria *Sporosarcina pasteurii* DSM 33 in the crack zones, the concentration of nutrient media was increased by 10 times compared to the recommended values by Shukla and Cameotra [8]. In this way, the influence of the diffusion processes on the MICP process was reduced. Although the selected nutrient media content was not optimal in the view of cultural heritage material (risk of NaCl occurrence), the nutrient medium content was optimal for the targeted activity of the selected bacteria [7]. Moreover, *S. pasteurii* DSM 33 belongs to a group of MICP agents who’s effective ureolytic activity is supported by the presence of ammonium ions in the matrix, and NH_4_Cl_2_ is a necessary part of the nutrient media [19]. On the other hand, due to the rapid and sudden changes in the pH value in the cell microenvironment, the nutrient medium involved substances with buffer effects, such as bicarbonate (NaHCO_3_). Bicarbonate is a natural buffer in groundwater and is the buffer of choice in microbiological laboratories. This substance also represents a source of required macroelements for bacterial viability.

### 2.4. Bio-Stimulated Healing Experiment

In order to assess the potential for crack closure in both the original historical mortar (HM) and the conservation mortar models (MM), two groups of materials were examined:

(1) Bio-samples: mortar samples (HM and MM) with the bio-activating agent and nutrient;
(2) Blank samples: mortar samples (HM and MM) with the nutrient only. These samples were used in order to ensure that the obtained crystals were not a consequence of the used nutrient or the development new crystal forms.

To eliminate potential microbial contamination, the samples were sterilised in an autoclave before the application of the bio-healing agent. This sterilisation process was performed to ensure that the results obtained in the laboratory could be attributed solely to the bio-activated healing process.

The cracks on the top surfaces of the samples were first treated with a sterile nutrient solution (300 µL) applied using the dropping technique. The bio-samples were then treated with a freshly prepared bacterial suspension (100 µL), while the blank samples were treated with 100 µL of sterile distilled water (instead of the bacterial suspension).

To maintain the constant humidity required for the incubation of the healing agent, the samples were kept in sterile distilled water (with approximately 1/3 of the sample’s height submerged) in Petri dishes for 150 days at 30 °C. The number of vegetative cells of *S. pasteurii* DSM 33 was periodically estimated by streaking on TSA with the addition of 20% urea every 7 days during the first month and every 30 days until the end of the incubation period. The pH value in the crack zone was also monitored at the same time as the bacterial viability using a HANNA HI 99,161 pH/temperature meter for surface measurement.

### 2.5. Evaluation of Healing Efficiency

The changes in the surface cracks of the MM and HM blank samples as well as the HM and MM bio samples were monitored every 7 days during the first 28 days of incubation and then every 30 days until the end of the 150-day period. As this was performed with a portable digital microscope, ViTiny Pro10-3 (ViTiny, Taylors, SC, USA), on the mortar samples kept in sterile distilled water, the obtained photos appeared blurred. The morphology of the formed calcium carbonate precipitate, after 28 and 150 days, inside the surface cracks of the MM blank and bio samples was examined by an SEM technique (without sample preparation). In order to study the chemical and structural properties of the CaCO_3_ at the end of experiment (150 days) as well as those of the sole precipitate taken out from inside the cracks, XRD and XRF techniques were used. XRD analysis was conducted on the powdered samples, while XRF analysis (with the same portable systems in the case of the historical mortars) was conducted directly on the treated and untreated area of the samples as well as on the precipitate taken out from inside the cracks. During the same period, the growth profiles of *S. pasteurii* DSM 33 were monitored. Namely, the number of vegetative cells of *S. pasteurii* DSM 33 was periodically estimated by powdering the tested samples and preparing serial dilutions. From the prepared dilutions, an aliquot of 100 μL was transferred onto TSA with the addition of 20% urea. This procedure was performed every 7 days within the first month and every 30 days until the end of the incubation period.

## 3. Results and Discussion

### 3.1. Historical Mortar Characteristics

**Figure 4:**
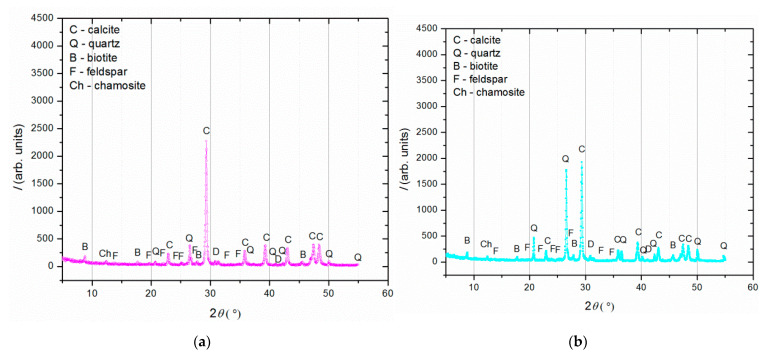

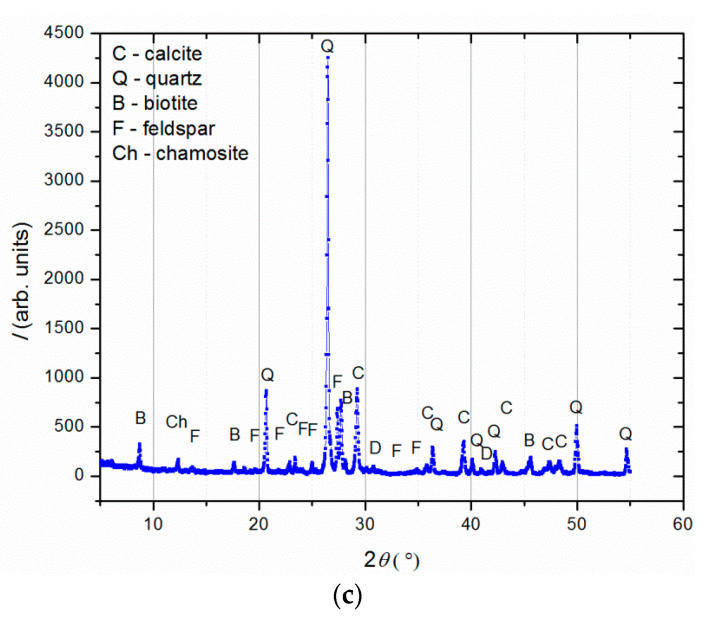
XRD analysis of (**a**) binder (fraction < 63 µm); (**b**) agglomerated binder/lumps (fraction > 710 µm); (**c**) aggregate (710–63 µm).

The results of the examination of the binder-to-aggregate ratio of the historical mortar by sieving and chemical analyses are given in Table 2 and Table 3, while the mineralogical compositions of the binder and the aggregate examined by XRD analysis are presented in Figure 4.

Based on the sieving and microscopic analyses (Table 2), it was revealed that the fractions larger than 710 µm represented agglomerated binder/lime lumps and the particles in the size interval 710–63 µm clearly indicated the morphology and colour characteristics of sand and crushed bricks, whereas the fraction smaller than 63 µm represented solely the binder. Based on the obtained results, the binder-to-aggregate ratio of the historical sample was calculated, while the chemical analyses (treatments with hydrochloric acid and sodium carbonate) determined the mass percentage of the sand and the crushed bricks in the aggregate.

Based on the sieving analysis, the aggregate-to-binder ratio was roughly calculated as 1.8:1, while the chemical analyses revealed that sand and crushed bricks took up 35.21% of the mixture. This implied that the remaining 64.79% was of a carbonate origin, including pozzolanic material (soluble SiO_2_) in the crushed brick as binder (Table 3).

XRD analysis (Figure 4) was performed on three separated fractions: (a) binder, <63 µm, (b) agglomerated binder, >710 µm and (c) aggregate, 63–710 µm. In the case of the examined fractions, the following mineral crystalline phases were detected: calcite [CaCO_3_], quartz [SiO_2_], biotite [(K(Mg,Fe)_3_(AlSi_3_O_10_)(F,OH)_2_], feldspar [KAlSi_3_O_8_-NaAlSi_3_O_8_-CaAl_2_Si_2_O_8_] and chamosite [(Fe_2_,Mg)_5_Al(AlSi_3_O_10_)]. In the case of the binder fraction (<63 µm), as expected, a significantly higher proportion of calcium carbonate (calcite) was identified, while in the case of the aggregate fraction (710–63 µm), a dominant presence of quartz was found. The identified calcite in the aggregate fraction was a consequence of limestone being used for the historical mortar preparation. The identified biotite was more significant in the case of the aggregate in relation to the binder fraction, which was expected since this mineral is usually present in river sands. Additionally, the slightly higher intensity of the identified chamosite in the aggregate relative to the binder fraction was a consequence of the already-identified particles of the crushed bricks. Finally, in the agglomerated binder/lumps (fraction >710 µm), the dominant mineral form, namely calcite, corresponded well to the composition of the binder fraction (<63 µm) except for the presence of quartz, which was intercalated in the agglomerated binder/lumps.

### 3.2. Compatible Conservation Mortars

Based on the results, the most promising model mortar (MM) was selected for conservation purposes (Table 1, Figure 2 marked as sample 5B) [6]. The composition of this mortar mixture was as follows: binder-to-aggregate volume ratio of 1 to 2 (slaked lime 1, crushed brick 0.2 as pozzolanic material, river sand 1.6 and milled limestone 0.2). This mixture was considered to be compatible since it exhibited lower a value of the average drilling force compared to the original historical mortar (HM) [4]. The total colour difference between the selected MM and HM (ΔE = 0.94) was lower than 3, which meant that the MM was visually highly compatible with the HM (the difference could not be detected with the naked eye) [18]. Moreover, the individual colour coordinates for the MM sample were approximately the same as that of HM (Table 4).

**Figure 5:**
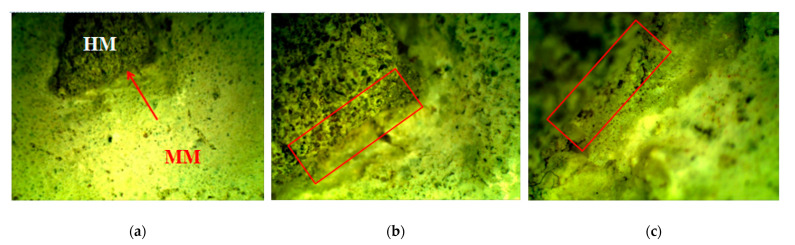
Stereo-optical microscopy: marked contact zone formation between original HM and chosen new mortar MM, magnification: (**a**) ×6.5; (**b**) ×20 and (**c**) ×40 [6].

The mutual interaction and cohesion between the original historical mortar (HM) and the laboratory-prepared conservation mortars (MM) was studied. A small fragment of the HM sample was put inside the most-promising new MM sample, as shown in Table 4. The interaction between the two samples was studied by using a stereo-optical microscope. During the carbonation/hydration process, a contact zone without cracks was formed between the original (HM) and the new (MM) sample. The morphology investigation, as shown in Figure 5, of the contact zone showed that the formed area had a satisfactory appearance (without cracks) [6,21].

In the next step, a sufficient number of samples of the selected MM mixture, as shown in Table 3, was prepared and artificially aged to allow further investigation. On these newly formed MM samples, surface cracks were created, and the samples with crack widths similar to or larger than the HM samples were chosen for the bio-stimulated crack closure.

### 3.3. Bio-Stimulated Crack Repair

The efficiency of the laboratory-prepared bio-stimulating crack-sealing system was continuously monitored by the analysis of the state of the surface cracks on the HM and MM blank and bio-samples.

#### 3.3.1. Growth Profiles of *S. pasteurii* DSM 33 on Bio-Samples

**Figure 6:**
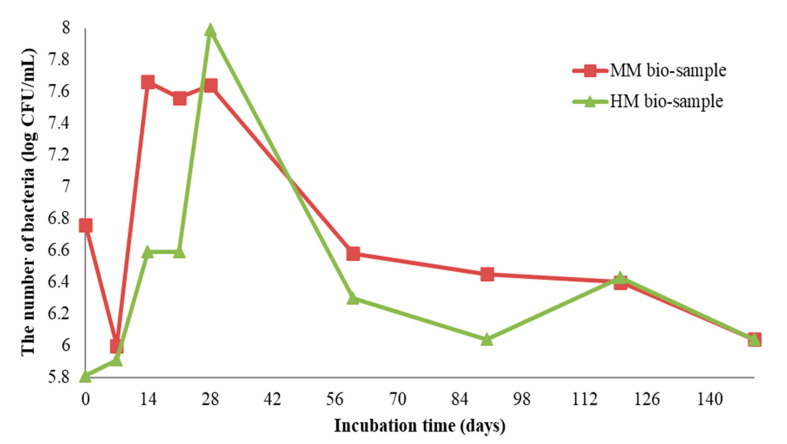
Enumeration of *S. pasteurii* DSM 33 on MM and HM bio-samples [6].

The growth profiles of *S. pasteurii* DSM 33 on the HM and MM bio-samples are shown in Figure 6. The initial number of bacterial spores of *S. pasteurii* DSM 33 in the suspension was 8.3 log CFU/mL (CFU—colony-forming unit). After the application of 100 μL of suspension onto/into the crack, the number of bacterial cells was above 5.8 log CFU/mL, dependent on the sample type (HM or MM). Within the first two months of incubation, the concentration of the cells was increased by at least 2 log CFU units. Decreasing trends for both the bio-samples were observed at the end of the incubation period. At the end of the experiment, the concentration of vegetative cells of *S. pasteurii* DSM 33 was above 6 log CFU units. According to the obtained results shown in Figure 6, it can be suggested that the observed concentration of vegetative cells could sustain the MICP process. Although the number of bacteria varied with time, it could be clearly concluded from Figure 6 that the cell concentration did not go below 6 log CFU/mL, even after 140 days. This number of cells was more than enough to encourage and sustain the MICP process and induce the precipitation of crystal forms in the crack zone during the experiment time [22].

**Figure 7:**
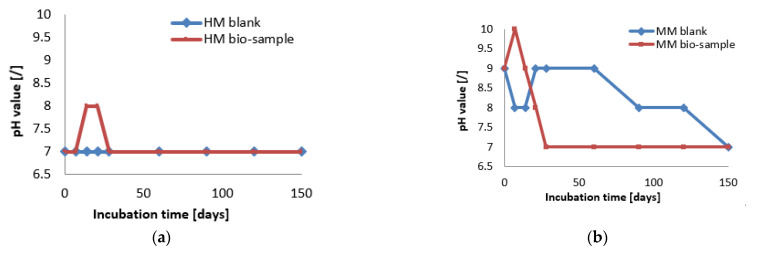
The pH monitoring of HM samples (**a**) and MM samples (**b**) [6].

At the start of the experiment, the HM samples had a lower pH value (approx. 7), while the MM samples were in the alkaline zone (approx. pH = 9). The mentioned pH values were in the pH zone for the bacterial growth and ureolytic activity of *S. pasteurii* (7–10) [20]. In the case of the HM samples, the pH value of the bio-sample did not change within the first 7 days, which was in correlation with the log (initial) growth phase of *S. pasteurii*. After the initial stagnation, the pH value of the HM bio-sample increased, suggesting the start of ureolytic activity. During the next 21 days, the pH value and the number of vegetative cells showed increasing trends (Figure 6 and Figure 7a). Besides a further increase in the vegetative cell number, the pH value of the HM bio-sample did not follow this trend and dropped to the initial pH value. These results suggested that ureolytic activity started, but the bacteria-induced precipitation slowed down due to the lack of free calcium ions in the historical samples. Moreover, it might be concluded that the ureolytic bacteria did not have optimal conditions for the MICP process in the HM samples, although the high number of vegetative cells indicated the possibility of bio-activation.

In the case of the MM samples, the pH value change in the bio-samples followed a trend similar to the bacterial viability, as shown in Figure 7. After a short decreasing period at the start, the pH value reached the initial level, and this value was maintained until 60 days of incubation. Due to a decrease in number of vegetative cells, pH value of the MM bio-sample also decreased until the end of incubation, reaching a final value of 7, as shown in Figure 7b. The HM blank sample remained stable during the incubation period, while the MM blank sample was affected by the continuous hydration process and had steep changes as well as a lower final pH value compared to the initial value.

#### 3.3.2. Digital Optical Microscopy

**Figure 8:**
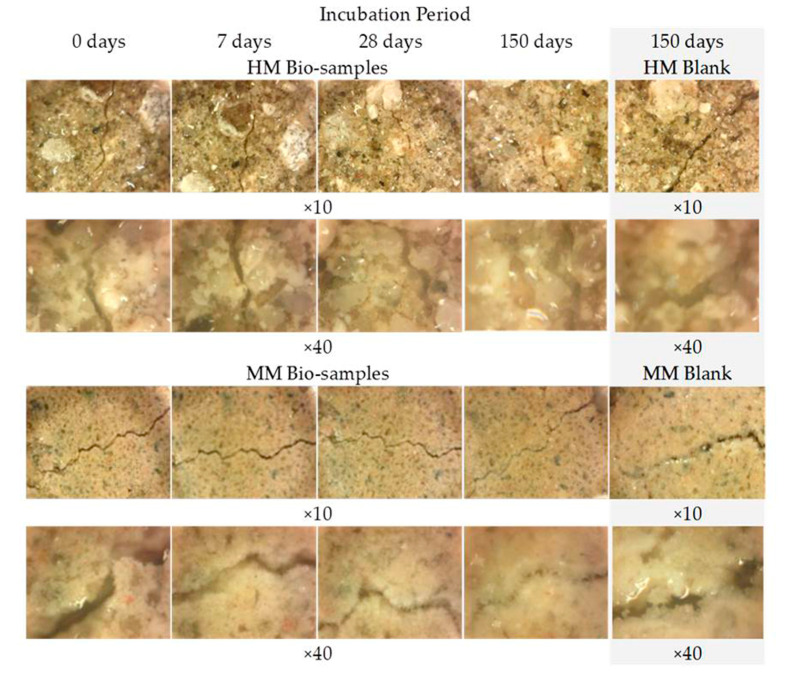
Microscopic images of HM and MM bio- and blank samples [6].

The images obtained by the digital optical microscope in the different phases of the experiment (0 days, 7 days, 28 days, 150 days) visibly indicated that the crack repair process was indeed obtained in the HM and MM bio-samples (Figure 8).

Comparing the microscopic images of the HM and MM bio-samples with the HM and MM blank samples after 150 days, the surface-crack reparation of the MM bio-samples was more pronounced and visible, as shown in Table 5 (the growth of needle-like crystals from the crack edges was identified). This could be explained by the fact that in the case of model mortars (MM) there were more calcium ions available for the bio-stimulated calcium carbonate precipitation, as at the time of the experiment, carbonization was still an on-going process. In the case of the historical bio-samples, the crack widths were similar or slightly lower compared to the original samples, and there was not a big difference in the closure ratio between 28th and 150th days of the experiment. Evidently, in the case of the “mature mortars”, much more than bio-stimulated carbonate sprouts is needed for the crack-healing process after 28 days. These results were in agreement with the results shown in Figure 6 and Figure 7. Namely, the ureolytic activity started, but the bacteria-induced precipitation slowed down due to a lack of free calcium ions in the historical samples. In order to obtain more information about the crack-repair effects in the case of the MM bio-samples, an SEM investigation was applied, while the historical samples were not examined further.

#### 3.3.3. SEM Analysis of Conservation Mortar Models

**Figure 9:**
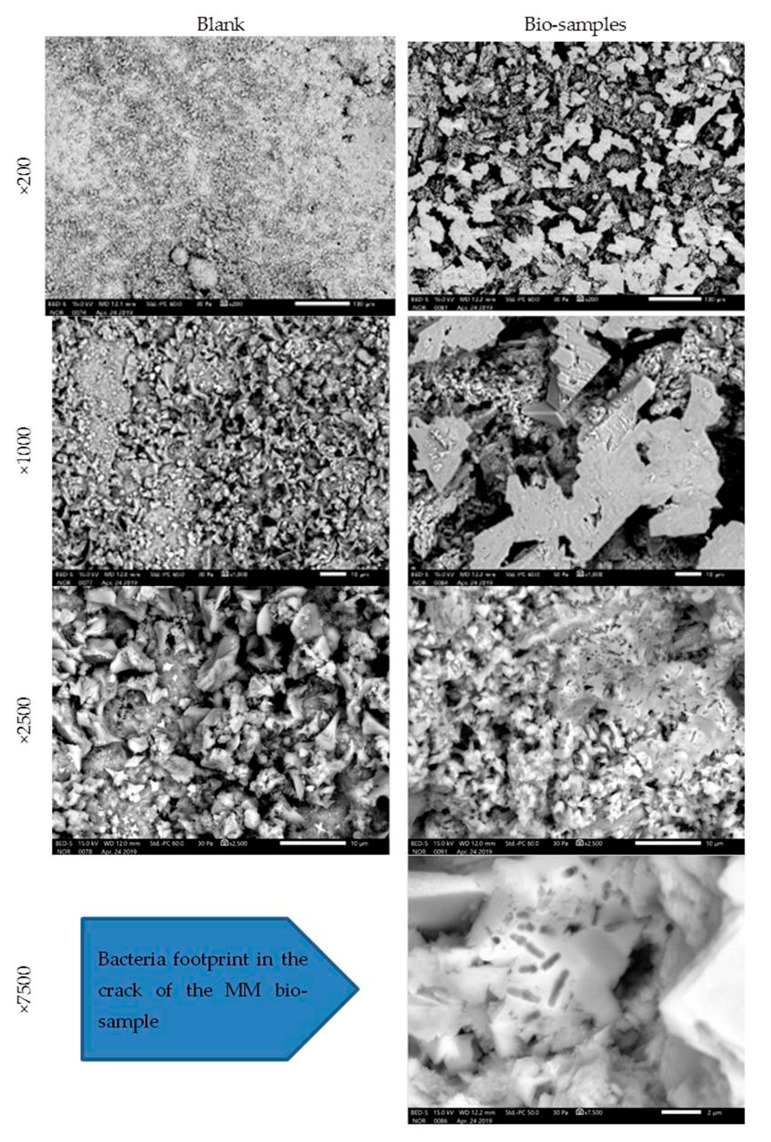
Microscopic images of MM bio- and blank samples after 28 days of incubation [6].

The MM bio-samples and blanks were analysed on the 28th day of incubation (Figure 9).

The obtained SEM images revealed evidence about the nature and structure of the precipitate. In the SEM images, the difference was easily observed between the blank and bio-samples of the MM after 28 days of incubation. The difference was in the clearly detectible presence of CaCO_3_ crystals in the precipitate of the bio-samples. Under a magnification of ×7500, the image of the MM bio-sample revealed a bacterial footprint (impressions) in the designed crack.

**Figure 10:**
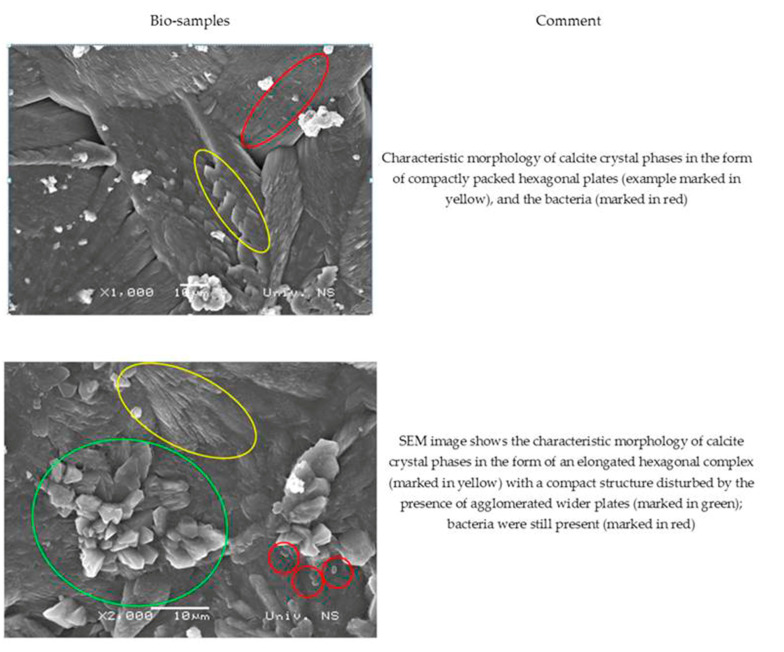
SEM analysis of MM bio-samples (precipitate formed in cracks) after 150 days of incubation [23].

The SEM images of the MM bio-samples after 150 days of incubation (Figure 10) reveal the effects of the advanced bacteria-stimulated formation of calcium carbonate in the cracks.

As shown in Figure 10, the SEM images of the MM bio-samples indicated the morphology of the surface crystal structures. The footprints of vegetative cells were not observed at the surface of the MM bio-samples. On the other hand, a sporogenic bacterial form (marked in red in both pictures) was detected around the already-formed CaCO_3_. Evidently, the identified bacterial spores stayed inactive from the beginning of the incubation period, and CaCO_3_ precipitation became a more important factor with increasing age in the humid environments.

#### 3.3.4. Comparative XRF Analysis of Conservation Mortar Models

An XRF investigation was performed at the end of experiment (150 days after the application) on the MM bio-sample in two areas: (1) in situ on the crack surface treated with the bio-agent and (2) in situ on the area not treated with the bio-agent. Additionally, the sole bacteria precipitate, taken from the same MM sample examined in situ on the crack surface, was also subjected to the XRF analysis. The comparative data are given in Table 6. The element concentrations were calculated based on the five spectra (five measuring points on the one sample) obtained by the XRF analysis on each area of the examined samples.

Based on the results presented in Table 6, it can be concluded that the bacterial activity in the crack of the MM sample increased the Ca concentration by 6% compared to the untreated area of the same sample. The XRF analysis of the sole bacterial precipitate sampled from the crack of the MM bio-mortar showed the presence of Ca (50.007%), confirming calcite precipitation.

#### 3.3.5. XRD Analysis

**Figure 11:**
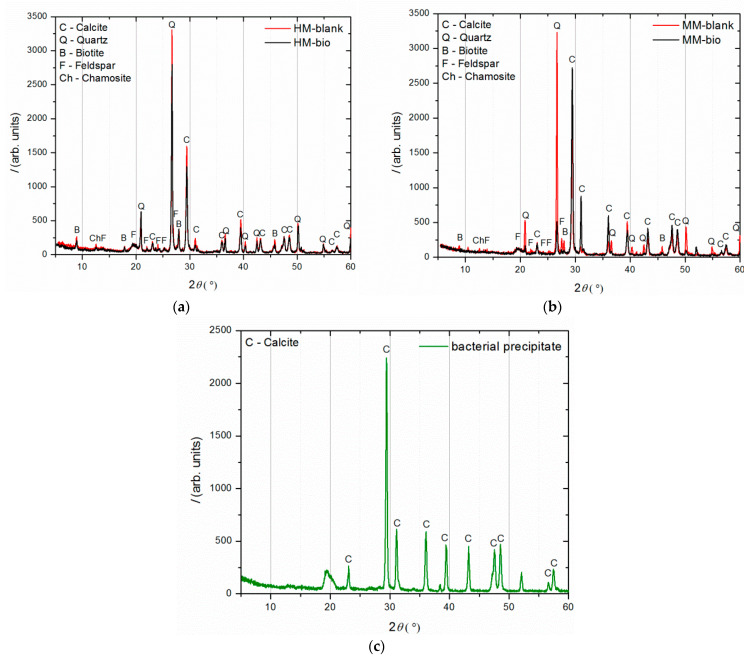
XRD analysis after 150 days of incubation: (**a**) HM blank and HM bio-samples comparative results, (**b**) MM blank and MM bio-samples comparative results, (**c**) sole bacterial precipitate.

The phase composition of the historical and model blank and bio-samples was examined by XRD analysis after 150 days of incubation. For each sample, the material subjected to these measurements was collected from the crack area in order to determine the crystalline structures formed during the healing process. Figure 11a shows the diffractograms of the historical mortars (HM blank and HM bio-sample), plotted together for comparison. Similarly, the XRD patterns belonging to the model mortars (MM blank and MM bio-sample) are shown in Figure 11b, while the pattern of the sole bacterial precipitate, taken from the MM bio-sample, is shown in Figure 11c.

Comparing the XRD patterns of HM blank and HM bio-samples (Figure 11a), there were no significant differences except for the intensity of all the diffraction peaks being reduced for the HM bio-sample. In contrary to this, comparing the MM blank and MM bio-samples (Figure 11b), the MM blank sample had a much higher quartz maxima than the MM bio-sample, while the MM bio-sample had a much more intense calcite maxima in comparison to the MM blank sample. This implied that strong calcite precipitation occurred in the MM bio-samples, in the region of the crack-healing area.

The comparison of the obtained results between the HM bio- and MM bio-samples confirmed that the bacteria-induced precipitation was limited in the HM samples because of the lack of free calcium ions. Namely, the “young” mortars with incomplete carbonation, as it was the case with the MM bio-samples, which were exposed to wet conditions, had a suitable quantity of free calcium ions inside ready for the healing processes [24]. The XRD analysis of the sole bacterial precipitate showed the presence of pure calcite, which confirmed that the increase in the calcite content in the healed MM bio-sample was a consequence of the bacterial activity. The unassigned peaks in Figure 11c belonged to the sample holder (a thin layer of 0.1 mm), which was used because there was a very little amount of the sample material.

## 4. Conclusions

The present study of bio-stimulated crack sealing in historical and conservation mortar samples showed a promising path towards the further investigation of bacteria-based systems for bio-stimulated healing effects on lime mortar surfaces (especially those from past conservation treatments). The experiments were performed in the laboratory using a bio-agent based on *Sporosarcina pasteurii* DSM 33 bacterial cultures. The monitored viability of the *S. pasteurii* DSM 33 cells during the 150-day experiment confirmed the potential of the chosen bacterial culture for the bio-stimulated healing of the selected substrates. A higher efficiency of the bio-stimulating crack healing was obtained in the case of the conservation mortar models compared to the original historical mortar samples. This can be explained by the observed lack of free calcium ions in the historical samples, both in the substrate itself as well as in the nutrient matrix used for the MICP process. Still, the large number of vegetative cells observed on/in the surface cracks of historical samples indicated that the bio-activation started, but it was interrupted due to the lack of free calcium ions.

The presence of CaCO_3_ crystals in the precipitate obtained in the conservation mortar models (MM) confirmed the activity of the developed bio-system. These findings were supported by the optical microscopy of the crack sealing as well as by the SEM and XRD analyses of the precipitate from the cracks. The absence of any crack-sealing effect in the blank samples of both the conservation mortar models (MM) and the historical samples (HM) stands to confirm that the selected bacterial culture, under the designed conditions, precipitated CaCO_3_ in/on the treated samples and stimulated surface crack sealing.

In the case of original mortar samples, the obtained results indicated that the “mature mortars” needed much more than bio-stimulated carbonate sprouts for the crack-healing process (after 28 days), as the ureolytic activity started, but the bacteria-induced precipitation slowed down due to the lack of free calcium ions.

These promising results can be used for the further investigation of surface healing of historical mortars. One pathway could be sought through upgrading the experiments with new bacterial carriers/nutrients, which will provide enough free calcium ions to maintain the bacterial activity and surface crack repair. This approach, if proven effective, could find its application in the preventive conservation of mortar structures to mitigate micro-cracks, maintaining aesthetic and functional characteristics. On the other hand, the obtained results could be used for the design of conservation bio-mortars by mixing the used bio-stimulating agent (*S. pasteurii* DSM 33 and nutrients) in fresh conservation mortar and testing its self-healing potential in the laboratory.

Based on the obtained results, our further research will explore the potential of reactivating dried samples (inactive, after 360 days) and examining if the bacteria can exhibit a new cycle of healing.

Moreover, our plan is to apply this bio-system in real environmental conditions. The main focus of this research will be the examination of the second phase of bio-stimulated crack closure. More precisely, CaCO_3_ precipitation connected with the age and level of humidity in a real environment will be primarily researched.

## Figures and Tables

**Table 1:** Mix design for preparation of model mortars (volume ratio).

Sample Mark	Component					
	River Sand	Crushed Brick	Milled Limestone	Quick Lime	Slaked Lime	Hydraulic Lime
1	2	0.1	-	-	-	1
2	1	0.2	0.8	-	1	-
3	1.5	-	0.3	-	1	-
4	1	0.2	1	-	-	1
4A	1.6	0.1	-	-	1	-
4B	1.7	0.2	0.2	-	1	-
5	1.8	0.2	-	-	1	-
5A	1.5	0.1	-	-	1	-
5B	1.6	0.2	0.2	-	1	-
5C	1.6	0.2	0.2	1	1	-
6	2.5	0.2	0.3	-	-	1.5
7	2	-	1	-	-	2
8	1.8	0.2	1	-	1	-
9	1.5	0.2	0.3	1	-	-
10	1.5	-	0.3	-	1	-
11	2	-	0.5	1	-	1
12	2	-	0.5	1.5	-	-
13	2	-	0.5	-	1	-

**Table 2:** Sieving analysis and optical microscopy of historical mortar sample, HM.

Particle Size (µm)	>1000	1000–710	710–500	500–315	315–250	250–125	125–63	<63
**wt.% in** **the sample**	15.26	9.88	10.97	15.11	8.96	23.87	5.27	10.67
** 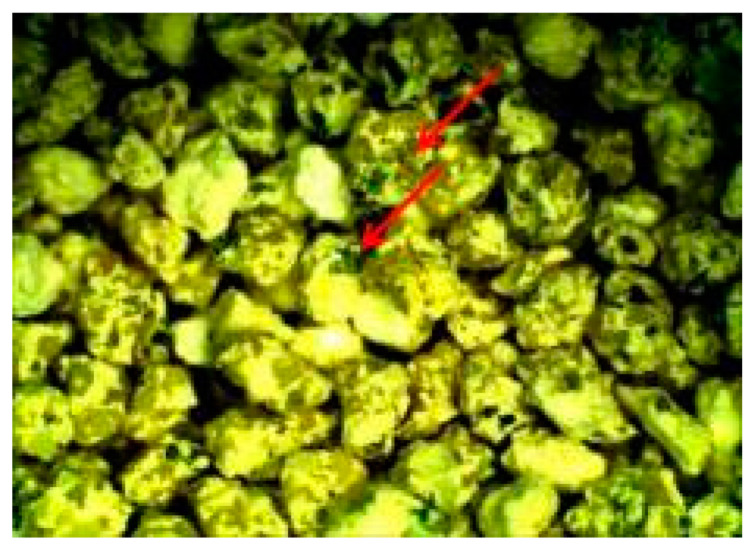 **				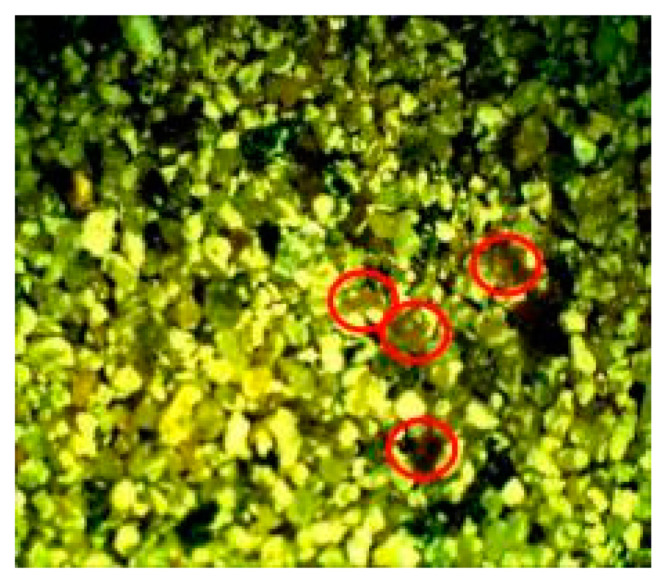				
**Fraction 1000–710 µm** **Agglomerated binder fraction;** **magnification ×45**				Fraction 250–125 µm Crushed brick particles marked; magnification ×20				

**Table 3:** Binder-to-aggregate ratio and chemical analyses of historical mortar sample, HM.

Binder-to-Aggregate Ratio		
	Binder	Aggregate
Characteristic	Fractions >710 µm and <63 µm	Fractions 710–63 µm
Quantity, wt.%	35.81	64.19
**Mass ratio**	**1.00**	**1.80**
**Volume ratio**	**1.00**	**2.10**
Chemical Analyses		
Treatment with HCl, wt.%		
Soluble residue		62.30
Insoluble residue		37.70
Insoluble residue treated with Na_2_CO_3,_ wt.%		
Soluble SiO_2_		6.58
Insoluble SiO_2_ (sand)		93.40

**Table 4:** Mechanical properties and spectrophotometry results [6].

Mortar Samples	Drilling Resistance Measurements (Drilling Force [N])		Colourimetry (Colour Coordinates and ΔE)			
	Average Value	Max. Value	L*	a*	b*	ΔE
Historical mortar (HM) Model mortar (MM)	0.5	0.6	79.08	1.45	10.77	0.94
	0.3	0.5	79.32	2.36	10.77	

CIELAB color coordinates (The International Commission on Illumination (CIE)): L* represents lightness from black to white on a scale of zero to 100; negative a* corresponds with green, positive a* corresponds with red, negative b* corresponds with blue and positive b* corresponds with yellow; **ΔE** Total colour diference.

**Table 5:** Measured values of the crack width.

Sample	HM Bio-Samples		MM Bio-Samples	
0 days, crack width, mm	Measured	0.045; 0.67; 0.045; 0.115; 0.090; 0.067	Measured	0.070; 0.094; 0.071; 0.098; 0.091; 0.085;
	Average	0.0715	Average	0.084
150 days, crack width, mm	Measured	0.084; 0.084; 0.036; 0.012; 0.024; 0.065	Measured	0.033; 0.020; 0.022; 0.062;
	Average	0.051	Average	0.034
Magnified detail of the measurement position				
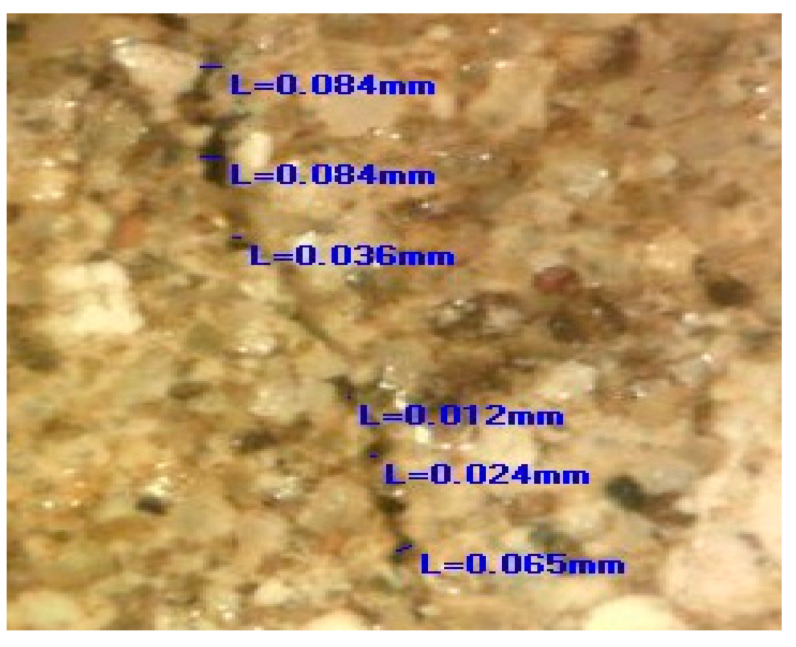 150 days HM sample			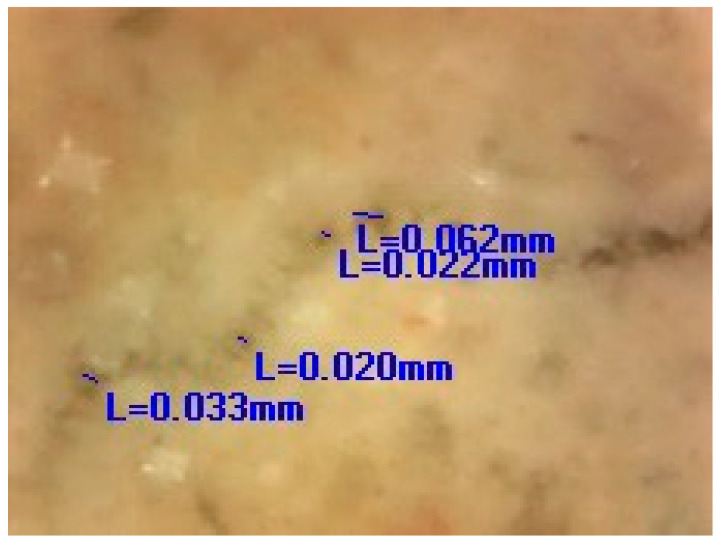 150 days MM sample	

**Table 6:** Results of the comparative XRF analysis.

	**MM Bio-Sample Crack Surface Treatedwith the Bio-Agent**	**MM Bio-Sample** **Untreated Area**	**Sole Bacteria** **Precipitate**
Element	Conc./%	Conc./%	Conc./%
Al	0.391	0.488	
Si	0.295	1.740	
P	0.318	0.276	
S	0.177	0.796	
Cl	0.001	0.053	1.461
K	0.637	0.655	
Ca	59.053	53.027	50.007
Ti	0.009	0.032	
Mn	0.005	0.004	
Fe	0.052	0.865	
Zn	0.018	0.034	
Sr	0.014	0.042	

## Data Availability

Not applicable.
